# The Influence of Remnant Cholesterol on Cardiovascular Risk and Mortality in Patients with Non-Functional Adrenal Incidentalomas and Mild Autonomous Cortisol Secretion: A Retrospective Cohort Study

**DOI:** 10.3390/jcm13195947

**Published:** 2024-10-06

**Authors:** Fernando Sebastian-Valles, Maria Jesús Fernández-Moreno, Iñigo García-Sanz, Natalia Fernanda Pascual Gómez, Víctor Navas-Moreno, Miguel Antonio Sampedro-Núñez, Monica Marazuela

**Affiliations:** 1 Endocrinology and Nutrition Department, Hospital Universitario de La Princesa, Instituto de Investigación Sanitaria de La Princesa, Universidad Autónoma de Madrid, 28006 Madrid, Spain; 2 Faculty of Medicine, Universidad Autónoma de Madrid, 28006 Madrid, Spain; mariajesus.fernandezm@estudiante.uam.es; 3 General Surgery Department, Hospital Universitario de La Princesa, Instituto de Investigación Sanitaria de La Princesa, Universidad Autónoma de Madrid, 28006 Madrid, Spain; garciasanzinigo@hotmail.com; 4 Clinical Laboratory Department, Hospital Universitario de La Princesa, Instituto de Investigación Sanitaria de La Princesa, Universidad Autónoma de Madrid, 28006 Madrid, Spain

**Keywords:** remnant cholesterol, adrenal incidentaloma, cardiovascular risk, autonomous cortisol secretion

## Abstract

**Background**: Increased cardiovascular risk has been described in individuals with adrenal incidentalomas. The aim of the present study is to assess the effect of remnant cholesterol (RC) on the cardiovascular risk and mortality of patients with adrenal incidentalomas. **Methods**: A retrospective cohort study was conducted with patients with adrenal incidentalomas between 2001 and 2024. One hundred thirty-seven patients (mean age of 61.2 ± 11.5 years; 56.6% women) with non-functioning adrenal incidentalomas and with mild autonomous cortisol secretion (MACS) (cortisol post-dexamethasone suppression test ≥1.8 µg/mL) were included. The patients were divided into two groups using 30 mg/dL as the cut-off for RC. Logistic regression models were used to study the impact of RC on major adverse cardiovascular events and mortality (MACEs). **Results**: Patients with RC ≥ 30 mg/dL exhibited a higher prevalence of type 2 diabetes mellitus (T2D) (*p* < 0.001), lower HDL-C (*p* < 0.001) and LDL-C (*p* = 0.025) levels, a higher frequency of treatment with statins (*p* = 0.032), and a higher rate of non-fatal major cardiovascular events (*p* = 0.038) and MACEs (*p* = 0.038). Patients with MACS showed no differences in RC or complications during the follow-up. The relative risk of high RC was 2.65 (1.04–6.77) for cardiovascular events and 2.27 (1.05–4.92) for MACEs, with *p* < 0.05 in both cases. The only variables independently affecting MACEs were age ([odds ratio] OR = 1.13 [*p* = 0.004]), female sex (OR = 0.20; *p* = 0.016), LDL-C (OR = 1.02; *p* = 0.029), and RC (OR = 1.06; *p* = 0.014). T2D and HDL-C were not independently associated with MACEs. **Conclusions**: RC ≥30 mg/dL in patients with adrenal incidentalomas was associated with a higher prevalence of T2D, lower HDL-C levels, and a higher risk of MACEs. MACS was not associated with RC or MACEs during the follow-up.

## 1. Introduction

Adrenal incidentalomas are adrenal lesions detected in imaging tests performed for indications different from suspected adrenal pathology [1]. The prevalence of adrenal incidentalomas is close to 3% in 50-year-old patients, rising to 10% in those with older ages [2]. These lesions encompass rare pathologies such as carcinomas, metastases, and pheochromocytomas, but they are mainly functioning or non-functioning adenomas. Incidentaloma evaluation requires ruling out the presence of malignancy and hormonal functionality (hypersecretion). These hormonal alterations include, among others, hypercortisolism, hyperaldosteronism, and pheochromocytoma. However, the most frequent hormonal alteration in adrenal incidentalomas is mild autonomous cortisol secretion (MACS), which occurs in up to 20% of all cases [2].

MACS refers to the presence of an increased cortisol concentration in the dexamethasone suppression test (DST) in which 1 mg of dexamethasone is administered at 11 p.m. and plasma cortisol is measured at 8 a.m. the next day; this increase is caused by a lack of cortisol suppression. Although numerous studies have investigated the threshold for MACS in DST, and cut-off values have been debated [3], current guidelines establish the cut-off point to be ≥1.8 µg/mL [3,4]. In addition to MACS, increased cardiovascular risk and other metabolic alterations have been observed not only in functioning (secreting) incidentalomas [5] but also in non-functioning lesions [6,7,8,9,10,11,12]. This heightened cardiovascular risk could be explained by alterations in cortisol secretion or metabolism that go unnoticed in routine diagnostic tests likely due to increased urinary excretion of cortisol metabolites [13]. Therefore, it has been proposed that hypercortisolism represents a continuum of hormonal and metabolic abnormalities with varying severity [14].

Remnant cholesterol (RC) refers to the cholesterol fraction found, in fasting conditions, in triglyceride-rich lipoproteins, including very-low-density lipoproteins (VLDL-C) and intermediate-density lipoproteins (IDL-C). It can be indirectly calculated by subtracting low-density lipoprotein cholesterol (LDL-C) and high-density lipoprotein cholesterol (HDL-C) from total cholesterol [15,16]. Recent studies indicate that RC levels may be considered as a novel cardiovascular risk factor. Higher plasma RC levels have been associated both with increased cardiovascular mortality in the general population, independent of HDL-C or LDL-C, and with an increased risk of atherosclerotic cardiovascular disease in healthy individuals [17] and a higher risk of ischemic heart disease, myocardial infarction, ischemic stroke, and all-cause mortality in the general population [15]. The cut-off point for RC concentration regarding its impact on cardiovascular risk has been established at 30 mg/dL [16,18,19].

Although adrenal incidentalomas are associated with cardiovascular risk, the impact of RC on this association remains to be studied. The aim of this study was to investigate the effect of dyslipidemia, specifically the novel factor RC, on the cardiovascular risk of individuals with adrenal incidentalomas.

## 2. Material and Methods

### 2.1. Study Design and Population

This is a retrospective cohort study. Data were collected from patients with adrenal incidentalomas treated at La Princesa University Hospital (Madrid, Spain) between January 2001 and January 2024. Radiological and laboratory tests at diagnosis were performed at our center. Cases of malignancy (adrenocortical cancer and metastases) and hormonal hypersecretion, including primary hyperaldosteronism, pheochromocytoma, and hypercortisolism (Cushing’s syndrome), were excluded. Hypercortisolism was diagnosed based on a suggestive clinical presentation (facial plethora, proximal myopathy, red/purple striae >1 cm wide, and easy bruising), unequivocally elevated urinary free cortisol excretion (threefold above the upper limit of the normality range of the assay), and lack of suppression in the 1 mg DST) [20,21,22].

### 2.2. Data Collection and Variables

The medical records of the patients included in this study were reviewed to collect data on the following variables: date of birth, sex, and date of diagnosis (date of the imaging test in which adrenal incidentaloma was first observed). Data at diagnosis were also collected for variables such as weight, height, body mass index (BMI), and personal history of hypertension, type 2 diabetes (T2D), osteoporosis, number of antihypertensive drugs, lipid-lowering drugs, and non-fatal ischemic cardiovascular events. Patients were divided into three categories according to their BMI, defined as being “normal weight” (BMI ≤ 25 mg/kg^2^), “overweight” (30 kg/m^2^ ≥ BMI > 25 mg/kg^2^), or “obesity” (BMI > 30 kg/m^2^).

Patients were recorded as having hypertension or T2D if these diagnoses were documented in their medical history or if they were receiving treatment for these conditions. Osteoporosis diagnoses were considered if they were documented in the medical history, if patients had densitometry results compatible with this condition, were undergoing treatment, or had experienced fractures with osteoporotic characteristics. Lipid-lowering treatment was classified into three levels of intensity based on the active ingredient and dose [23].

Additionally, personal histories of non-fatal cardiovascular events (defined as ischemic heart disease, stroke, or visceral or limb ischemia) at diagnosis and at the last available visit, along with the date of the event, were recorded. Strokes documented in the medical history, transient ischemic attacks, amaurosis fugax, and signs of stroke in imaging tests with compatible clinical symptoms were included. Furthermore, the following variables were defined: major cardiovascular events (including ischemic heart disease, stroke, and visceral and limb ischemia events from diagnosis to the last follow-up date) and the composite endpoint major adverse cardiovascular event (MACE), including major cardiovascular events and mortality. Other variables collected included lesion size, date of surgery (if applicable), date of last follow-up visit, and cause and date of death (if applicable).

Biochemical assessment at diagnosis included glycated hemoglobin (HbA1c), serum creatinine, total cholesterol levels, and LDL-C and HDL-C concentrations. RC was calculated as the difference between total cholesterol and the sum of HDL-C and LDL-C [16]. Regarding hormonal assessment, serum basal cortisol and urinary free cortisol were measured at diagnosis, and all patients underwent a DST [4,24]. Cortisol measurement in the laboratory was performed using Chemiluminescent Microparticle Immunoassay (CMIA, Alinity I, Abbot). Additionally, MACS was defined as plasma cortisol ≥1.8 µg/mL in the DST [3,4]. Glycated hemoglobin was routinely determined using liquid chromatography (ADAMS A1c HA8180 V ARKRAY^®^). Cholesterol was analyzed using an enzymatic method (Alinity C Cholesterol Reagent Kit, Abbott).

Subsequently, patients were divided into two groups (low RC and high RC) based on whether they had RC at diagnosis <30 mg/dL or ≥30 mg/dL [18]. This study was approved by the Drug Research Ethics Committee of La Princesa University Hospital (registration number 5489) on 8 February 2024.

### 2.3. Statistical Analysis

Data obtained from each patient were anonymized and entered into a database. Statistical analysis was performed using Stata/BE 17 (StataCorp LLC), licensed in 2021, and free R software. Continuous variables were reported as mean ± standard deviation or median and interquartile range depending on their distribution, and categorical variables were reported as numbers of events and percentages. The Shapiro–Wilk test was used to assess the normality of variables. Mann–Whitney U or Kruskal–Wallis tests and two-tailed t-test or ANOVA were used to evaluate group differences for variables with non-normal and normal distributions, respectively. The association between variables was assessed using Spearman’s Rho.

Logistic regression models were performed to study the composite endpoint MACE. The models were constructed while adjusting for age, sex, RC, LDL-C, HDL-C, MACS, HbA1c, adrenalectomy, overweight, obesity, lipid-lowering therapy, and previous non-fatal ischemic cardiovascular events. Statistical significance was set at *p* < 0.05.

## 3. Results

### 3.1. Patient Characteristics

**Figure 1:**
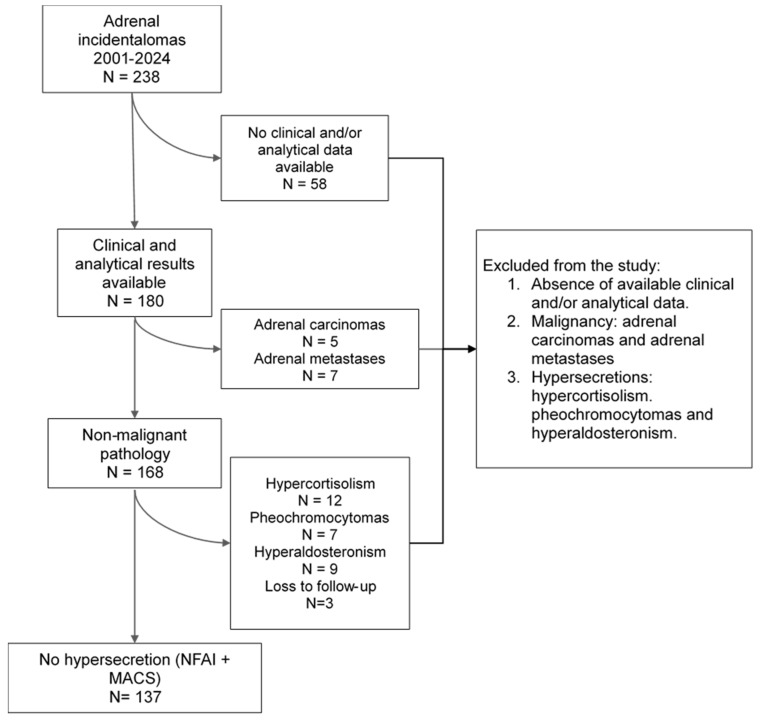
Recruitment flowchart. NFAI: non-functioning adrenal incidentalomas. MACS: mild autonomous cortisol secretion.

A total of 137 subjects were included in this study. A patient recruitment flowchart is shown in Figure 1. The mean age of the patients was 62.5 ± 10.8 years, and 80 (58.4%) were female. Eighty-two (59.9%) patients were undergoing antihypertensive treatment, and fifty-five (40.1%) patients were undergoing lipid-lowering therapy. Forty-six (33.6%) subjects met the MACS criteria, and eleven (8.03%) had a history of non-fatal ischemic cardiovascular events. Thirty-two patients (23.4%) had a history of malignant cancer.

Subjects were classified as having high RC or low RC according to whether the RC values were ≥30 mg/dL or <30 mg/dL, respectively. The sociodemographic and clinical characteristics of both groups are shown in Table 1. No statistically significant differences were observed in most of these characteristics between both groups. Only the percentage of patients with T2D and lipid-lowering treatment were significantly different in the group of patients with high RC compared to those with low RC (47.1% vs. 14.6%; *p* < 0.001), and (32.1% vs. 55.9%; *p* = 0.032), respectively. Conversely, the concentrations of HDL-C (46.0 ± 8.5 mg/dL vs. 61.8 ± 14.1 mg/dL; <0.001) and LDL-C (106.1 ± 41.5 mg/dL vs. 121.9 ± 34.7 mg/dL; *p* = 0.025) were significantly lower in patients with high RC. All comparisons between high and low RC are shown in Table 1.

**Figure 2:**
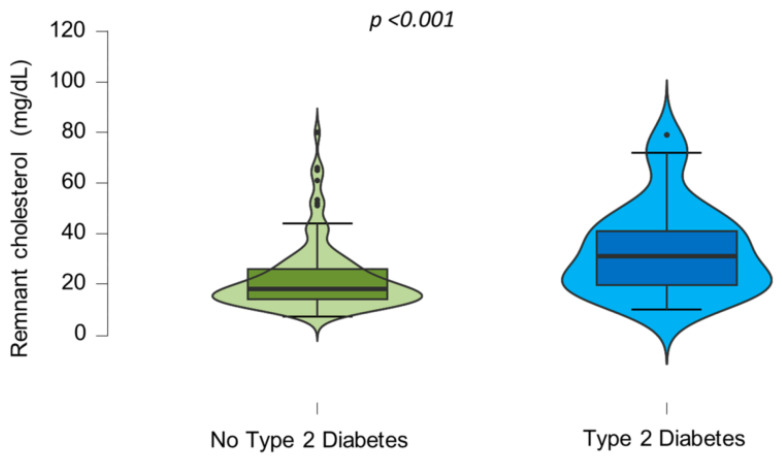
Correlation between RC levels and HDL levels at diagnosis. HDL-C high-density lipoprotein cholesterol. Moderate statistically significant negative correlation is observed between HDL-C levels and remnant cholesterol (Rho −0.489 *p* < 0.001).

The variables associated with RC (HDL-C and T2D) were further studied to better characterize this association. A Spearman correlation coefficient analysis showed a moderately inverse correlation between RC and HDL-C (Figure 2) (Spearman’s Rho = −0.489).

**Figure 3:**
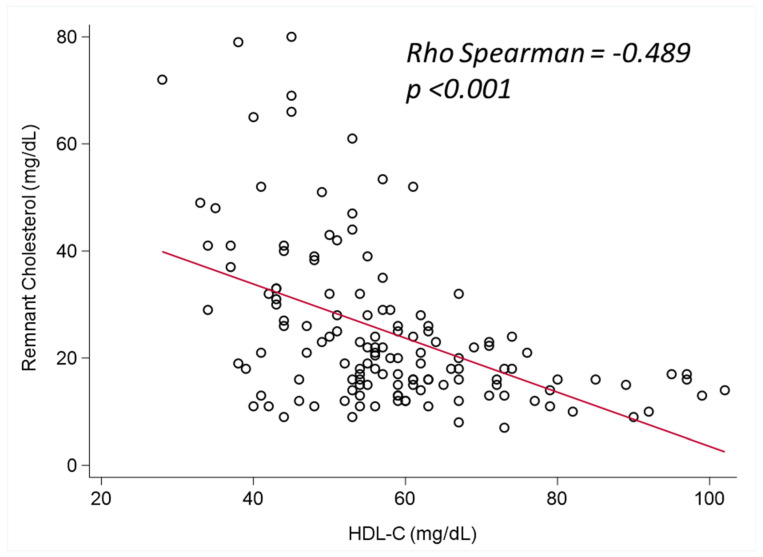
Increased levels of remnant cholesterol in patients with type 2 diabetes. Violin plots depicting remnant cholesterol levels in subjects with type 2 diabetes and in those without the disease. Remnant cholesterol is significantly higher in patients with type 2 diabetes. Data were analyzed with Mann–Whitney U test.

Additionally, the RC levels were significantly higher in individuals with T2D compared to those without this disease (*p* < 0.001) (Figure 3).

Lastly, we investigated whether HDL-C levels and the presence of T2D were independently associated with RC levels. This was confirmed through a multiple regression model, which determined that both factors were statistically significant predictors. Specifically, HDL-C had a β coefficient of −0.453 (*p* < 0.001) and T2D a β coefficient of 6.83 (*p* = 0.014).

### 3.2. Cardiovascular Events and MACEs

Subsequently, the impact of RC on non-fatal cardiovascular events and the composite endpoint MACE was studied. The relative risk of high remnant cholesterol (RC ≥ 30 mg/dL) was 2.65 (95% CI 1.04–6.77) (*p* = 0.038) for non-fatal cardiovascular events and 2.27 (95% CI 1.05–4.92) (*p* = 0.038) for MACEs.

Finally, a logistic regression model was performed to verify whether RC levels independently influence MACEs. Additionally, the model was adjusted for relevant variables such as age, sex, HbA1c, HDL-C, LDL-C, MACS, surgery, lipid-lowering treatment, overweight, obesity, and history of cardiovascular events prior to adrenal incidentaloma diagnosis. This analysis showed that age, sex, LDL-C, and RC were the only variables independently influencing MACEs, with *p* < 0.05 (Table 2). The odds ratios were 1.17 for age (95% CI 1.05–1.29), 0.12 for female sex (95% CI 0.03–0.52), 1.06 for RC (95% CI 1.01–1.11), 1.02 for LDL-C (95% CI 1.00–1.05), and 7.36 for previous history of a cardiovascular event (95% CI 1.11–48.55). However, HbA1c, MACS, adrenalectomy, overweight, obesity, and HDL-C were not independently associated with an increased risk for MACEs.

## 4. Discussion

RC is a novel risk factor associated with cardiovascular complications and mortality in various diseases and in the general population [15,17,18]. Our study found that RC was independently associated with major cardiovascular events and mortality in patients with non-functioning adrenal incidentalomas and with MACS.

The literature supports an association between adrenal incidentalomas, both functional and non-functional, and increased cardiovascular risk [5,6,7,8,9,10,11,14]. Our study reinforces these findings by providing additional information on lipid disorders, specifically high RC. Our results highlight this parameter as a potential factor implicated in the elevated cardiovascular risk and mortality observed in subjects with adrenal incidentalomas irrespective of the presence of MACS. Adrenal incidentalomas without MACS are associated with alterations in hormonal metabolite excretion, which may explain the high cardiovascular risk in these patients compared to the general population [13,25]. Additionally, RC has been associated with numerous cardiovascular risk surrogates, such as poorer glycemic control [19], higher rates of retinopathy [26], diabetic nephropathy [27,28], and coronary calcification [29]. Our study suggests that RC may contribute to the impact of these factors given its association with major cardiovascular events and mortality [15,17,18].

This study did not demonstrate an association between MACS at diagnosis and major cardiovascular events and mortality. Our findings align with other studies suggesting that the differences between MACS and non-functioning incidentalomas are small, indicating that these conditions likely represent a continuum of comorbidity risk rather than well-differentiated pathologies [12]. In addition, the relationship between lipid fractions and cortisol secretion is complex. Our study did not find a relationship between RC and MACS, which is consistent with other studies that failed to observe an association between cortisol secretion and lipid metabolism alterations [30,31]. However, further analyses using more precise metabolomics techniques will be necessary to investigate the potential association of urinary metabolite secretions with the lipid profile and risk of cardiovascular events in these populations. These analyses will also allow us to determine whether the effect on cardiovascular risk is direct or if it occurs through a potential form of mediation or interaction between the lipid profile and hormonal secretion.

Although the main findings of our study are consistent with the current literature, there are discrepancies in some respects. Whereas higher levels of RC have been associated with an increased risk of chronic kidney disease [27] and a decreased risk of cancer [15], these associations were not observed in our study. This is likely due to the small sample size and the low prevalence of chronic kidney disease. Additionally, surgery has been shown to improve cardiovascular risk factors in both MACS and non-functioning adrenal incidentalomas [32]. However, in the multivariate analysis of our study, the odds ratio for surgery suggested an increased cardiovascular risk, although this association was not statistically significant and could be attributed to a less favorable clinical profile for patients treated with surgery compared to those who are untreated. Randomized clinical trials or observational studies employing propensity scores will be necessary to evaluate the impact of adrenal surgery on lipid control and major cardiovascular events during follow-up.

This study has certain limitations. It is a retrospective, observational study with a small sample size, and it therefore lacks sufficient statistical power to demonstrate causality and elaborate survival curves. Additionally, the longitudinal evolution of autonomous cortisol secretion was not studied despite knowing that some features such as bilateralism and a size larger than 3 cm in incidentalomas increase the risk of hormonal secretion progression [2,33]. Another major limitation of this study is the use of CMIA as a technique for measuring plasma cortisol instead of liquid chromatography–tandem mass spectrometry (LC-MS/MS), as CMIA has lower sensitivity and specificity and provides less precise results, which usually contributes to overestimated measurements [34,35]. Therefore, among the subgroup of patients classified as having MACS in our study, there could be false positives, leading to underestimation in the results. Our study used 1.8 µg/mL as a cut-off in the DTS for the MACS assessment. Although this criterion follows international guidelines [4], it is important to acknowledge that dexamethasone suppression has shown limited predictive value for morbidity [24]. Another limitation of this study is the lack of radiological density in Hounsfield units due to its absence in older radiology reports. The limited sample size and the low proportion of events during the follow-up also precluded a separate multivariable analysis of individuals with MACS and non-functioning adrenal incidentalomas [36,37]. Finally, the results were not compared with a control group without adrenal pathology because this was not the focus of this study. Further research in studies including controls without incidentalomas are necessary to fully elucidate the effect of RC levels on major cardiovascular events in patients with non-functioning adrenal incidentalomas and MACS.

## 5. Conclusions

RC is an independent risk factor associated with cardiovascular disease and mortality in patients with non-functioning adrenal incidentalomas and MACS regardless of variables such as age, sex, or plasma cortisol levels after the dexamethasone suppression test. Additional multicenter prospective studies are needed to more comprehensively assess the cardiovascular impact of lipid alterations in individuals with adrenal incidentalomas.

## Figures and Tables

**Table 1:** The characteristics of the patients included in this study according to whether they exhibited high or low remnant cholesterol.

Variable	Total Sample n = 137	Remnant Cholesterol <30 mg/dL n = 103	Remnant Cholesterol ≥30 mg/dL n = 34	*p* Value
Age (years)	62.5 ± 10.8	62.7 ± 11.0	62.1 ± 10.6	0.523
Sex (woman)	80 (58.4%)	62 (60.2%)	18 (52.9%)	0.457
Cancer (%)	32 (23.4%)	23 (22.33%)	9 (26.5%)	0.621
Surgery (%)	14 (10.2%)	8 (7.8%)	6 (17.7%)	0.100
Size (mm)	26.9 ± 18.9	25.8 ± 17.7	30.5 ± 22.1	0.298
BMI (Kg/m^2^) Normal weight Overweight Obesity	28.2 ± 5.9 34 (24.8%) 55 (40.1%) 48 (35.0%)	27.6 ± 6.0 28 (27.1%) 45 (43.7%) 30 (29.1%)	30.1 ± 5.1 6 (17.6%) 10 (29.4%) 18 (52.9%)	0.074 0.086
Diabetes mellitus (%)	31 (22.6%)	15 (14.6%)	16 (47.1%)	<0.001
Dexamethasone suppression test (ug/mL)	1.92 ± 1.60	1.82 ± 1.35	2.25 ± 2.0	0.277
Mild autonomous cortisol secretion	46 (33.6%)	32 (31.1%)	14 (41.2%)	0.279
HTA (%)	57 (41.6%)	41 (39.8%)	16 (47.1%)	0.457
Number of antihypertensive drugs 0 1 2 3 ≥4	82 (59.9) 21 (15.3%) 17 (12.4%) 12 (8.8%) 5 (3.7%)	65 (63.1) 17 (16.5%) 10 (9.7%) 6 (5.8%) 5 (4.85%)	17 (50.0%) 4 (11.8%) 7 (20.6%) 6 (17.6%) 0	0.100
Total cholesterol (mg/dL)	201.2 ± 40.4	201.3 ± 38.1	204.3 ± 37.4	0.591
HDL (mg/dL)	57.9 ± 14.5	61.8 ± 14.1	46.0 ± 8.5	<0.001
LDL (mg/dL)	117.9 ± 36.9	121.9 ± 34.7	106.1 ± 41.5	0.0249
HbA1c (%)	5.9 ± 1.0	5.7 ± 0.8	6.5 ± 1.3	0.001
Intensity of lipid-lowering treatment No treatment Low intensity Moderate intensity High intensity	85 (62.0%) 8 (5.8%) 33 (24.1%) 11 (8.0%)	70 (67.9%) 6 (5.8%) 22 (21.4%) 5 (4.9%)	15 (44.1%) 2 (5.9%) 11 (32.4%) 6 (17.7%)	0.032
eGFR (mL/min)	87.9 ± 19.2	88.6 ± 19.3	84.9 ± 17.7	0.470
eGFR < 60 mL/min	6 (4.4%)	5 (4.9%)	1 (2.9%)	0.636
Osteoporosis	13 (9.5%)	10 (9.7%)	3 (8.8%)	0.879
Previous non-fatal ischemic cardiovascular events	11 (8.03%)	9 (8.8%)	2 (5.9%)	0.595

eGFR (Estimated Glomerular Filtration Rate), HbA1c (Glycated Hemoglobin), HDL-C (High-Density Lipoprotein Cholesterol), HTA (Arterial Hypertension), BMI (Body Mass Index), LDL-C (Low-Density Lipoprotein Cholesterol). Categories according to BMI: normal weight, BMI ≤ 25 mg/kg^2^; overweight, 30 kg/m^2^ ≥ BMI > 25 mg/kg^2^; obesity, BMI > 30 kg/m^2^.

**Table 2:** Logistic regression model assessing the independence of associations influencing the composite endpoint MACE.

Variable	Odds Ratio	95%CI	*p* Value
Age (years)	1.17	1.05–1.29	0.002
Sex (woman)	0.12	0.03–0.52	0.005
HbA1c (%)	1.96	0.98–3.89	0.054
Remnant cholesterol (mg/dL)	1.06	1.01–1.11	0.019
HDL-C	1.00	0.95–1.06	0.898
LDL-C	1.02	1.00–1.05	0.026
MACS	0.75	0.20–2.75	0.673
Adrenalectomy	1.55	0.23–9.84	0.652
Lipid-lowering therapy	3.17	0.74–13.6	0.119
Previous non-fatal ischemic cardiovascular events	7.36	1.11–48.55	0.038
Overweight	0.26	0.05–1.38	0.115
Obesity	0.48	0.08–2.76	0.408

CI, confidence interval; HDL-C, high-density lipoprotein cholesterol; LDL-c, low-density lipoprotein cholesterol; MACE, major adverse cardiovascular events; MACS, mild autonomous cortisol secretion. Age, LDL-C levels, remnant cholesterol levels, and history of non-fatal cardiovascular events (*p* < 0.05) are independently associated with composite endpoint MACE.

## Data Availability

F.S.-V and M.M. are the guarantors of this work and, as such, had full access to all of the data in this study and take responsibility for the integrity of the data and the accuracy of the data analysis.
